# Transcriptome Analysis of Chinese Chestnut (*Castanea mollissima* Blume) in Response to *Dryocosmus kuriphilus* Yasumatsu Infestation

**DOI:** 10.3390/ijms20040855

**Published:** 2019-02-15

**Authors:** Cancan Zhu, Fenghou Shi, Yu Chen, Min Wang, Yuqiang Zhao, Guomin Geng

**Affiliations:** 1 Institute of Botany, Jiangsu Province and Chinese Academy of Sciences, Nanjing 210014, China; zhucancan858@163.com (C.Z.); 15150530195@163.com (Y.C.); wm120624@163.com (M.W.); zhaoyuqiang123@126.com (Y.Z.); 2 College of Forestry, Nanjing Forestry University, Nanjing 210037, China; fhshi406@163.com

**Keywords:** transcriptome, Chinese chestnut, *Dryocosmus kuriphilus*, infestation, gall formation

## Abstract

Chinese chestnut (*Castanea mollissima* Blume) can be infested by *Dryocosmus kuriphilus* Yasumatsu, resulting in gall formation and yield losses. Research on the control of gall wasps using genomics approaches is rarely reported. We used RNA-seq to investigate the dynamic changes in the genes of a chestnut species (*C. mollissima* B.) during four gall-formation stages caused by *D. kuriphilus*. A total of 21,306 genes were annotated by BLAST in databases. Transcriptome comparison between different gall-formation stages revealed many genes that were differentially expressed compared to the control. Among these, 2410, 7373, 6294, and 9412 genes were differentially expressed in four gall-formation stages: initiation stage (A), early growth stage (B), late growth stage (C), and maturation stage (D), respectively. Annotation analysis indicated that many metabolic processes (e.g., phenylpropanoid biosynthesis, secondary metabolism, plant–pathogen interaction) were affected. Interesting genes encoding putative components of signal transduction, stress response, and transcription factors were also differentially regulated. These genes might play important roles in response to *D. kuriphilus* gall formation. These new data on the mechanism by which *D. kuriphilus* infests chestnuts could help improve chestnut resistance.

## 1. Introduction

Chestnuts are economically important nuts and timber-producing trees that include the Chinese chestnut (*Castanea mollissima*), Japanese chestnut (*Castanea crenata*), European chestnut (*Castanea sativa*), and American chestnut (*Castanea dentata*) [1,2]. Chinese chestnut (*Castanea mollissima* BL) is a major cultivated species producing edible chestnuts in China [1]. However, *C. mollissima* has been affected by major diseases and pests which have caused significant losses. The chestnut gall wasp, *Dryocosmus kuriphilus (Hymenoptera Cynipidae)*, which is native to mainland Asia, was introduced to Japan, North America, and Europe and has become a significant pest of cultivated chestnuts [3]. *D. kuriphilus* can prevent normal plant growth and cause a progressive loss of photosynthetic biomass [4]. Hence, it is important to investigate effective strategies to combat this pest.

Insect galls are composed of plant tissues that develop in response to insect-derived stimuli, and present inducer-specific phenotypes and patterns of tissue differentiation [5]. Gall development is closely related to tissue redifferentiation, and the formation of a gall-specific cell type is dependent on the identity of a host plant cell [6]. Gall formation is composed of three well-distinguished phases: initiation, subsequent growth, and final maturation [7]. Galls can alter the physiology, morphology, anatomy, development, and chemistry of the plant host in ways that are beneficial to the pest [6,8,9], but the mechanism of gall development is poorly understood [10]. Understanding the host response to insect infestation is needed to develop more effective control strategies.

In the battle between plants and agents, plants have evolved immune systems that can recognize potentially intrusive agents and initiate effective defense responses [11]. Host changes in response to insect invasion could shed light on the strategies used by the insect [12]. For example, gall-inducing insects can suppress plant defenses for their own benefit [13]. Therefore, looking at the responses elicited by insects may identify key plant functions that are targeted during plant manipulation by the insect. The current knowledge of the effects on plants induced by gall-inducing insects has been reviewed [14]. However, a different host might have different responses to different gall-making insects [2]. Plant hormones are key to gall development [15]. Cooper et al. reported that the induction of jasmonates in plant tissues could affect aspects of insect-induced gall development and maintenance [3]. Additionally, the insect can also manipulate the nutritional and defensive biochemical traits in chestnut galls [16]. The effect of insects on plants and the strategies employed by insects to remodel the cell content and structure of plant tissues at their feeding sites were well reviewed by Giron et al. [14]. However, the interaction between Chinese chestnuts (*Castanea mollssina* Blume) and *D. kuriphilus* still need further study.

Genomic tools, such as transcriptome analysis, can aid in the identification of resistance genes and the development of blight-resistant chestnut [2]. High-throughput RNA-seq has been used to study genetic and molecular mechanisms underlying host plant resistance to pests [10,17,18,19,20]. Additionally, an investigation of the host transcriptome change in response to pests can facilitate the isolation of resistance genes, which can improve the efficiency of resistant varieties breeding by genetic engineering. However, an integrated analysis of the host transcriptome has not been reported for chestnut–insect interactions.

Hence, in this study, we investigated changes in the Chinese chestnut gall transcriptome induced by *D. kuriphilus*. The gall-formation stages mentioned above are easy to differentiate visually and are natural sampling points for investigating the interaction between pest and host plant tissues during gall development. However, in this study, considering the gall grows rapidly during the growth stage, we hypothesize that the gene expression of the plant host will also change dramatically during the growth stage. Hence, we divided the growth stage into early growth stage and late growth stage visually. Finally, we focused on four gall-formation stages: initiation (A), early growth (B), late growth (C), and maturation (D) stage to investigate the dynamic changes of gene expression of the Chinese chestnut (*Castanea mollssina* Blume). Differentially expressed genes (DEGs) analysis enabled identification of a large number of candidate pathogen-response genes in Chinese chestnut for use in studying pathways involved in resistance to *D. kuriphilus*. This host–insect interaction may be important in breeding programs for resistant genotypes. Also, information about host responses is useful to advance our understanding of the mechanism by which *D. kuriphilus* affects other species of trees.

## 2. Results

### 2.1. Gall Formation Process

**Figure 1:**
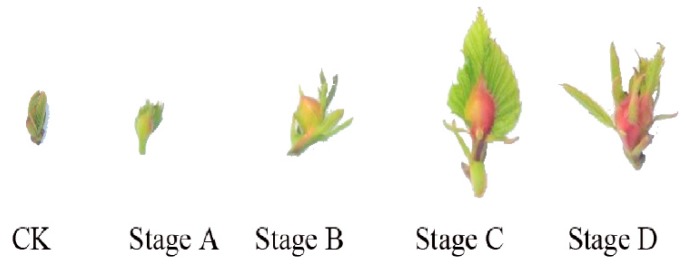
Development stages of gall formation produced by *D. kuriphilus* infestation. CK: A healthy leaf used as control.

Phenotype changes of the different development stages of gall formation by *D. kuriphilus* are shown in Figure 1. Galls are small at the initiation stage (Gall_ A). During the growth stages (Gall_ B and Gall_ C), the gall is larger and more visible. Finally, galls lignify and take on a deep red appearance at the maturation stage (Gall_ D).

### 2.2. Sequencing, Assembly, and Annotation

To obtain a global overview of the Chinese chestnut transcriptome during leaf gall formation, a total of 15 cDNA libraries were constructed and sequenced on a Illumina Hiseq 2500 platform. After removing adaptors and low-quality sequences, a total of 295.14 million clean reads were obtained. On average, 23.94 million clean reads were obtained from each sample (Table S1). The percentages of Phred-like quality scores at the Q20 level (error probability of 1%) ranged from 98.2 to 98.4% (Table S1). Among the 15 samples, 67.96–74.03% of the clean reads were mapped to the reference genome, and 59.63–65.78% of clean reads were uniquely mapped (Table S1). The saturation curves of 15 RNA-seq samples (genes with FPKM ≥ 0.01) estimated that the gene coverage began to show saturation when approximately more than 20 million clean reads were aligned (Figure S1). The number of average clean reads from the 15 samples was 23.94 million, which exceeds the saturation threshold. Detailed information on the RNA-Seq data is listed in Table S1. These data indicate high sequencing quality and sequencing depth sufficient for transcriptome coverage.

To test sample repeatability, we calculated the correlation coefficient between the samples. The correlation coefficient in the repeat group reached 0.99 or higher (Figure S2), indicating that the differential expression analysis between different groups is reliable. A total of 21,695 genes were detected in the transcriptome by the Hisat2 program, including 17,681 genes that were FPKM ≥ 1. Among these, 21,306 genes were annotated by at least one of the seven databases, and 9.56% (2074) of genes annotated in all of the databases.

### 2.3. Analysis of Differentially Expressed Genes (DEGs) in Chinese Chestnut Leaf During the Leaf Gall Formation

**Figure 2:**
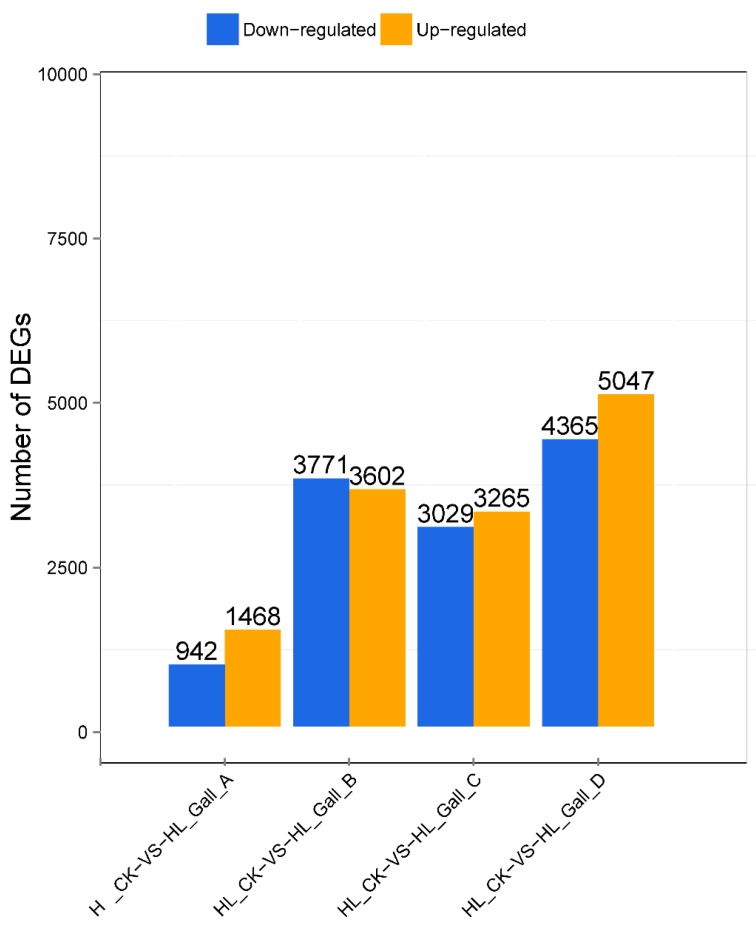
Number of differentially expressed genes during different gall-formation stages.

Using the criteria of Fold change ≥2 and divergence probability ≥0.8, a total of 2410, 7373, 6294, and 9412 genes were differentially expressed in the four gall-formation stages compared to the control (Figure 2). The four time points could be distinguished by the differentially expressed genes. The least and most differential genes were detected respectively in stages A and D compared to the control, indicating an increasing effect on plant leaves as the gall developed. In the A phase, the differential gene was significantly less than other stages. This indicated that the initial growth stage (B) of the gall was the initial large response of the plant leaf to the gall damage. During this period, the gall may have begun to absorb the nutrients in the leaves, and the leaf damage increased.

**Figure 3:**
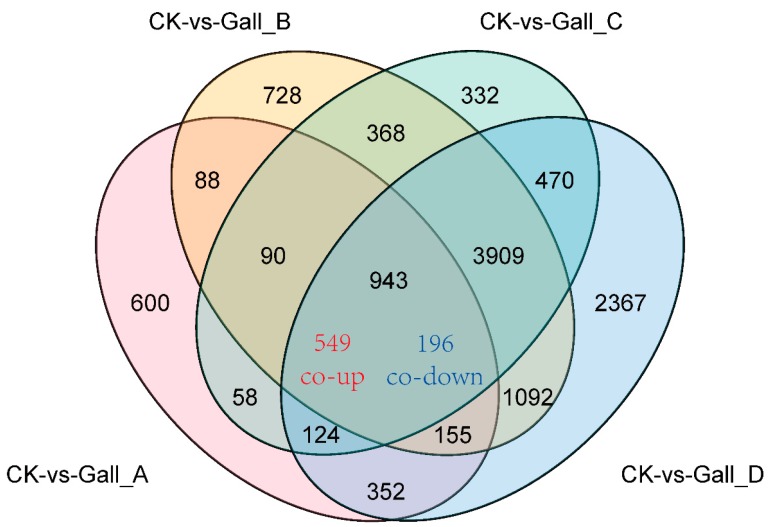
A Venn diagram comparing significantly different expressed genes at different gall-formation stages.

The Venn diagram (Figure 3) shows that there were 549 co-upregulated genes and 196 co- downregulated genes at A, B, C, and D stages compared to the control. The numbers of upregulated genes were greater than downregulated genes under gall stress at both time points, suggesting gall damage may active many stress-response activities.

**Figure 4:**
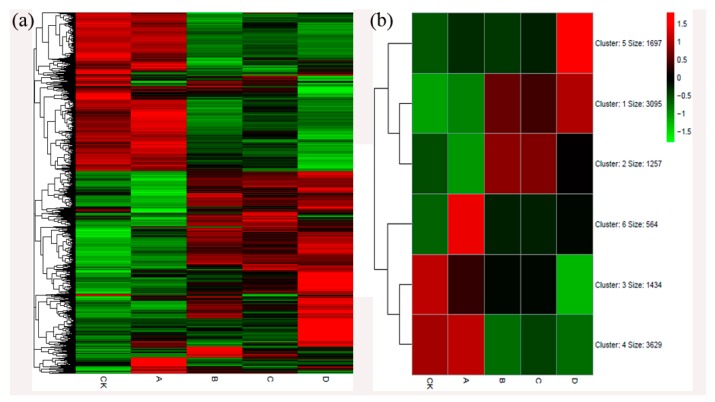
Heat maps of differentially expressed genes (DEGs). (**a**) Heat maps of DEGs; (**b**) Cluster analysis of DEGs.

To further explore the trends of these differentially expressed genes, we used the R package pheatmap to draw heat maps of DEGs (Figure 4a). DEGs were clearly divided into two categories with K-mean = 6 (Figure 4b). The first large group of genes was expressed more in the control and stage A, and the second largest group had higher expression in the B/C/D stage. This result also demonstrated that the response of the leaves to gall damage started to increase in the B stage.

**Figure 5:**
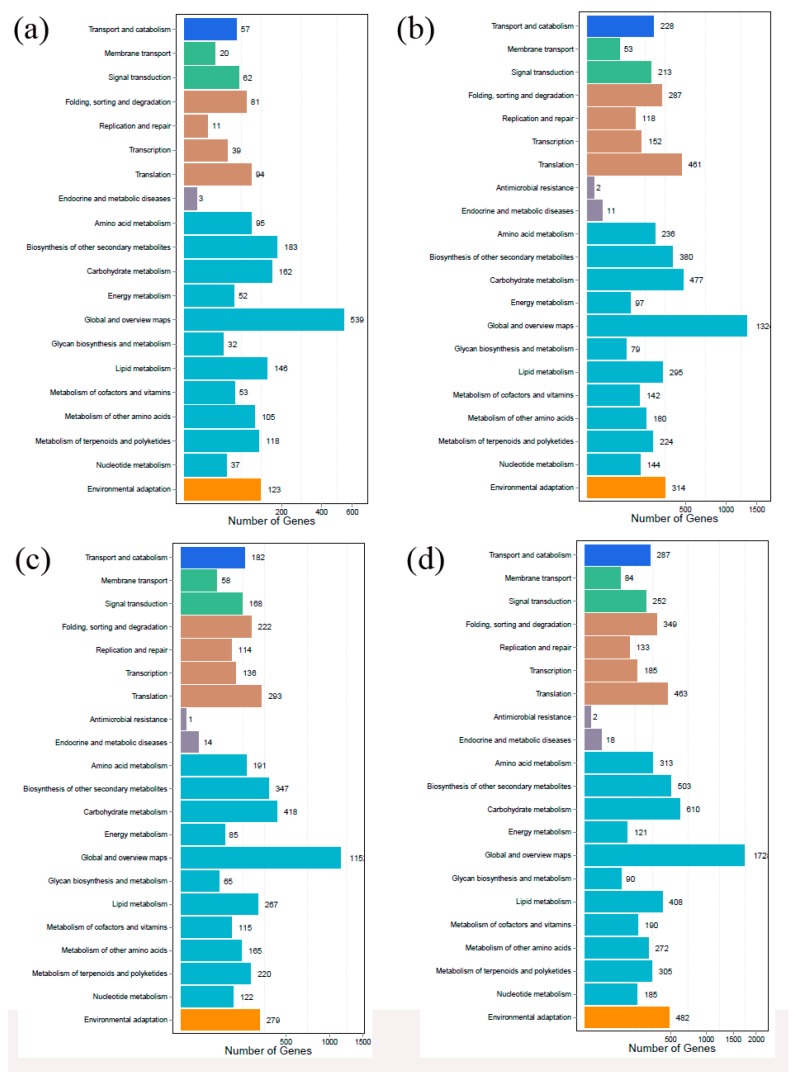
KEGG analysis of DEGs. (**a**) KEGG analysis of DEGs at stage A; (**b**) KEGG analysis of DEGs at stage B; (**c**) KEGG analysis of DEGs at stage C; (**d**) KEGG analysis of DEGs at stage D.

All of the DEGs were BLAST against the KEGG Ontology (KO) database. At stage A, 1035 annotated DEGs were classified into 130 biological pathways belonging to 20 KEGG categories (Figure 5a). At stage B, 2949 annotated DEGs were classified into 135 biological pathways belonging to 21 KEGG categories (Figure 5b). At stage C, 2465 annotated DEGs were classified into 131 biological pathways belonging to 21 KEGG categories (Figure 5c). At stage D, 3764 annotated DEGs were classified into 134 biological pathways belonging to 21 KEGG categories (Figure 5d).

**Figure 6:**
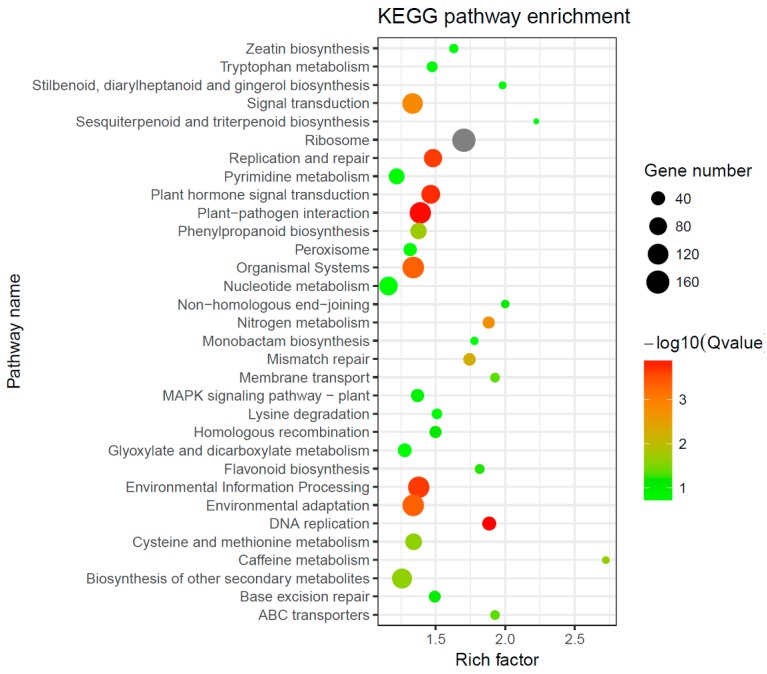
KEGG enrichment analysis of DEGs.

We also used the Benjamini and Hochberg (BH) multiple test correction to analyze KEGG enrichment for all of the DEGs in four stages (*p* < 0.05, FDR < 0.05). Enrichment of “plant–pathogen interaction”, “Plant hormone signal transduction”, and “phenylpropanoid biosynthesis” was found with a threshold of false discovery rate (FDR) < 0.05 (Figure 6). Furthermore, sesquiterpenoid and triterpenoid biosynthesis; stilbenoid, diarylheptanoid, and gingerol biosynthesis; flavonoid biosynthesis; and peroxisome were also found with a threshold of *p* < 0.05 (Figure 6).

### 2.4. DEGs Involved in Signaling and Transcription Were Altered during Leaf Gall Formation

**Figure 7:**
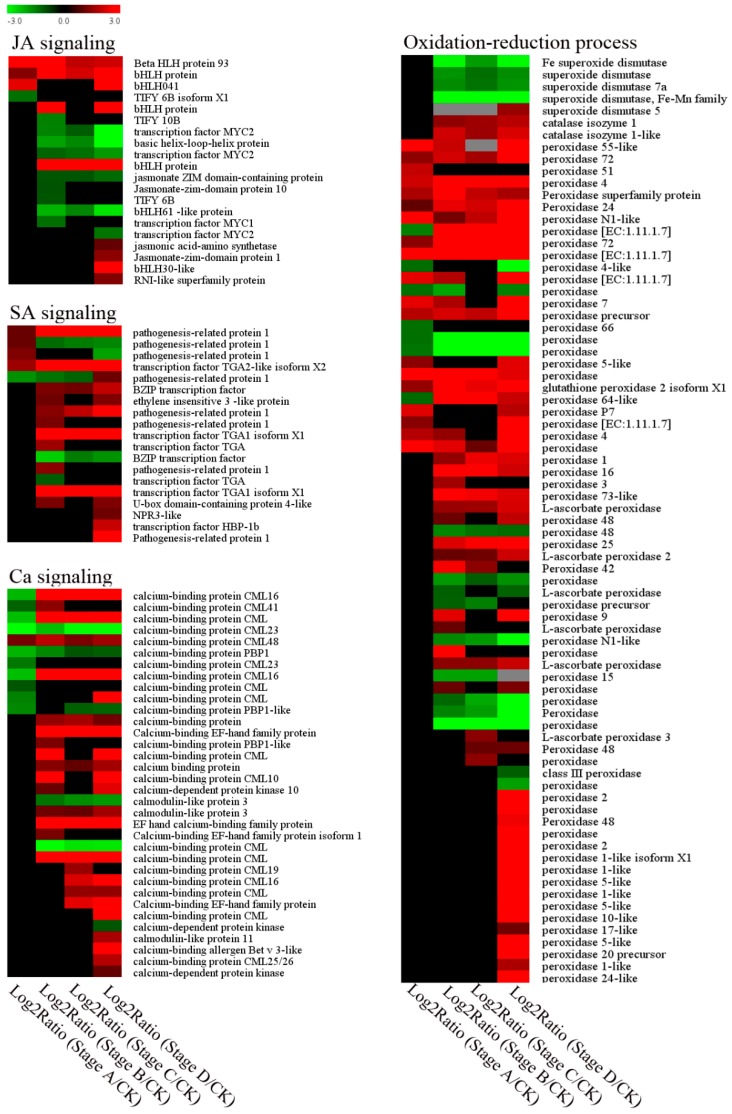
Expression profiles of DEGs involved in JA, SA, Ca^2+^ signaling and oxidation–reduction process.

Plant hormone signaling was altered in the infested *C. mollissima*. In JA signaling, there were four genes encoding a transcription factor that were differentially expressed at stage A, with three upregulated and one downregulated (Figure 7). Similarly, genes related to SA signaling were also significantly induced at the initiation stage of gall formation (Figure 7). Besides hormone signaling, the results also indicated that the majority of the Ca^2+^-signaling-related genes were upregulated (Figure 7).

Genes encoding transcription factors were also identified with different expressions in the control and the infested host. Eleven *WRKY* genes were upregulated while only one gene was downregulated at the initiation stage of gall formation (Excel S1).

### 2.5. DEGs Involved in Oxidation–Reduction Process Were Altered by D. kuriphilus Infestation

Reactive oxygen species (ROS) metabolism-related genes showed different expression patterns during gall formation. Four *SOD* genes were significantly downregulated from stage B to stage D, while only one *SOD* gene was upregulated at stage D (Figure 7). In contrast, two *CAT* genes were upregulated along with gall formation (Figure 7). Another gene family involved in the oxidation–reduction process was *peroxidase*. We identified 70 *peroxidase* genes, including *glutathione peroxidase* and *L-ascorbate peroxidase*. The majority of these were upregulated, especially stage D (Figure 7). Some were involved in the response to stress (Excel S1). Besides peroxidase, we also identified many genes involved in peroxisome. Six *respiratory burst oxidase* genes were all upregulated in the chestnuts (Excel S1).

### 2.6. Regulation of Secondary Metabolism during Gall Formation by D. kuriphilus

**Figure 8:**
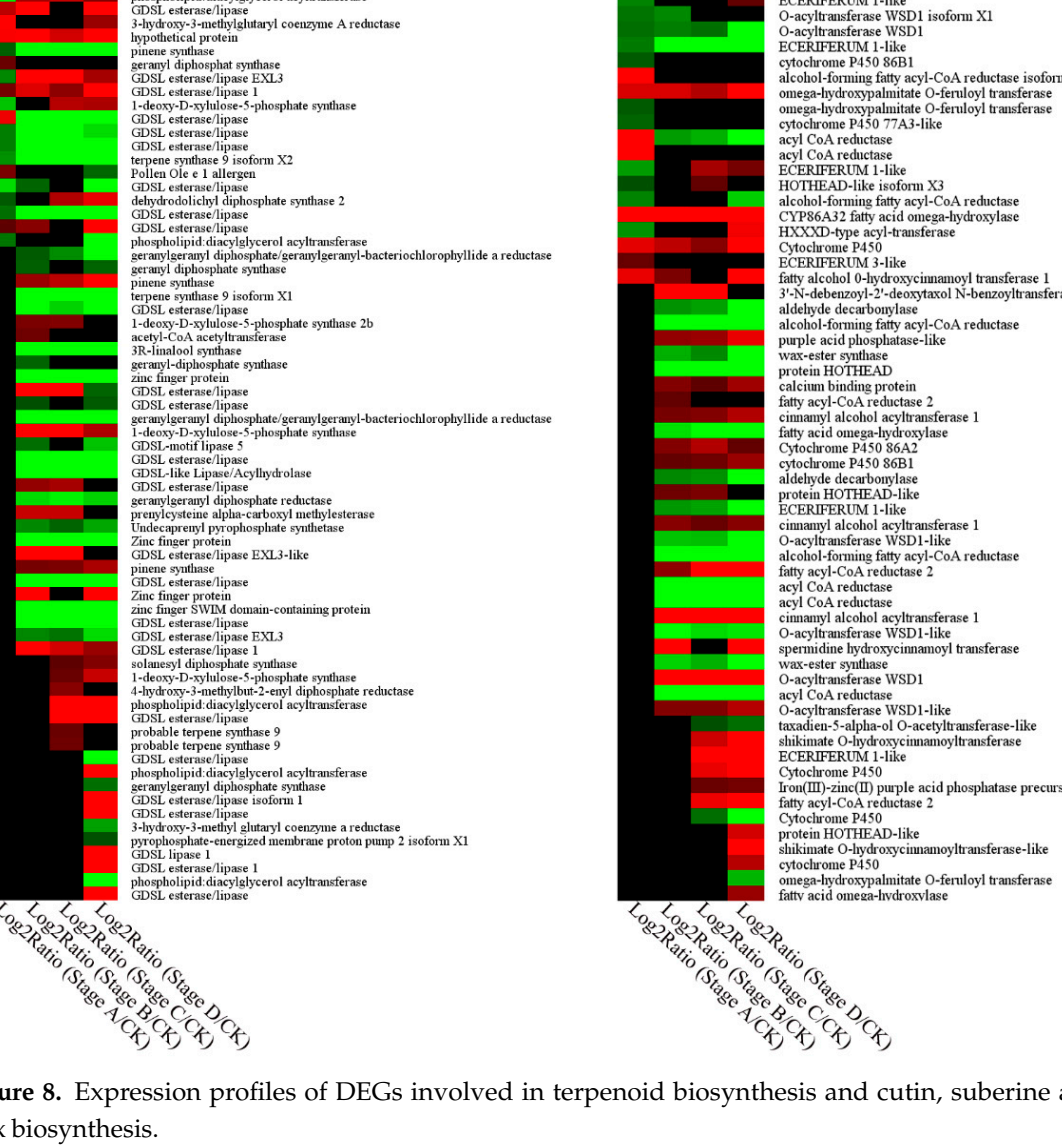
Expression profiles of DEGs involved in terpenoid biosynthesis and cutin, suberine and wax biosynthesis.

Of the genes involved in secondary metabolism, 72 were involved in terpenoid biosynthesis, and they were differentially expressed during gall formation. These genes were mainly *GDSL esterase/lipase*, and most of them were downregulated during the gall formation process (Figure 8). Genes encoding terpene synthase showed significant differential expression in response to *D. kuriphilus* infestation. One gene was downregulated during the whole process of gall formation. One *terpene synthase* was downregulated from the growth stage (stage B) to mature stage (stage D), and two were slightly upregulated only at stage C (Figure 8). The expression level of *geranylgeranyl diphosphate synthase* also decreased at stage D.

We also identified 64 genes that were involved in cutin, suberine, and wax biosynthesis (Figure 8). Among them, two *wax ester synthase* genes were significantly downregulated. In addition, three *O-acyltransferase WSD1* were also identified, and these decreased during the whole gall-formation process (Figure 8). The *cytochrome P450* gene was also identified with different expression patterns, including seven upregulated and three downregulated.

Transcripts responsible for phenylpropanoid biosynthesis were active during gall formation on the plant host. All the *phenylalanine ammonia-lyase* genes identified in this study were found to be upregulated during the gall-formation process (Excel S1). Four *caffeoyl-CoA O-methyltransferase* (*CCOAOMT*) genes were also upregulated, with only one downregulation. One *cinnamyl-alcohol dehydrogenase* (*CAD*) and two *shikimate O-hydroxycinnamoyltransferase* were identified with upregulation during the whole gall formation process. Four *caffeic acid O-methyltransferase* (*COMT*) were identified, with three upregulated and only one downregulated.

### 2.7. Validation of Some DEGs

**Figure 9:**
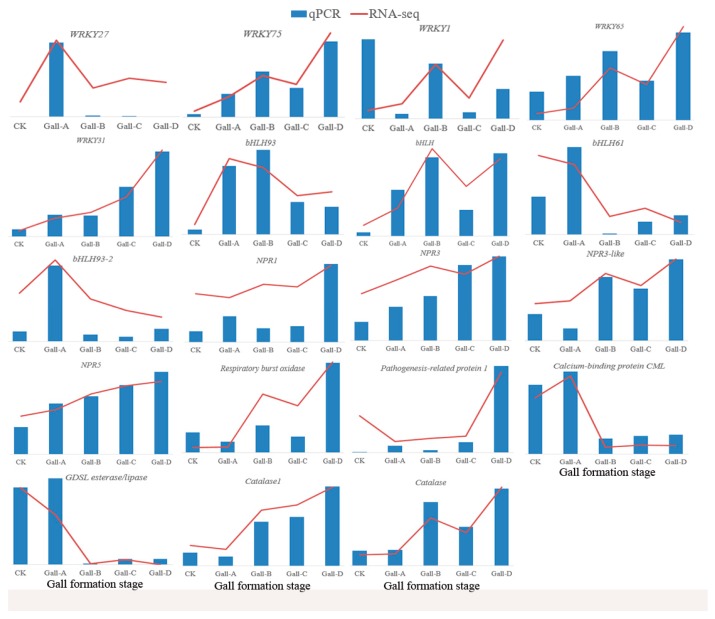
Conformation of the RNA-seq data by qRT-PCR.

Quantitative real-time PCR (qRT-PCR) was conducted to validate some of the DEGs. Nineteen genes were selected according to the following criteria: (a) significantly differential expression among different gall-formation stages and (b) previously reported to be potentially involved in a plant defense response such as genes involved in transcription factors, signaling, responses to stress, and secondary metabolism. Results of the qRT-PCR analysis were globally consistent with RNA-seq data (Figure 9). Only three genes (*WRKY1*, *bHLH61*, and *pathogenesis-related protein1*) showed different expression patterns between these two approaches at the first two gall stages. RNA-seq analysis was mainly used for high-throughput screening and indicated the global gene expression patterns of different samples. Hence, the mismatch of the expression profile shown by the RNA-Seq and qRT-PCR experiments always occurred. Additionally, the qRT-PCR results based on specific gene primers should be more reliable.

## 3. Discussion

*D. kuriphilus* is one of the most damaging pests of *Castanea* spp. [21]. *D. kuriphilus* galling prevents flower and shoot development, and can even contribute to tree mortality [3]. Here, we investigated the effect of gall formation by *D. kuriphilus* on Chinese chestnuts based on the transcriptome approach. As shown in Figure 1, the host tissue changed dramatically during different gall-formation stages. RNA-seq results also indicated that the four stages could be distinguished by the differentially expressed genes. The number of DEGs increased between the initiation stage and the mature stage, suggesting an increased effect on plant leaves as the gall formation (Figure 2). This study increases our knowledge of the genetic regulation of gall formation on chestnuts.

### 3.1. Signal Transduction and Transcription Factors Were Affected in Chestnuts during the Gall Formation by D. kuriphilus

Plant defense against biotic stress can be regulated by the signal transduction pathway. Plant hormones such as jasmonic acid (JA) play a central role in this pathway [3,22]. However, the role of JA in response to galling insects can be different in different plant hosts. For example, a tephritid maggot (*Eurosta solidaginis* Fitch) and a gelechiid caterpillar (*Gnorimoschema gallae-solidaginis* Riley), do not induce JA in their Solidago host plants [23], nor does the Hessian fly (*Mayetiola destructor* Say) attack wheat (*Triticum aestivum* L.) [24]. In contrast, Nabity et al. found that the gene expression for JA (LOX)-defense signaling increased in grapes in response to *Daktulosphaira vitifoliae* infestations [25]. Additionally, previous studies have indicated that JA treatments benefit gall wasps by increasing gall size and defenses [3]. In the present study, several *bHLH* transcription factors were significantly upregulated according to RNA-Seq and qRT-PCR results (Figure 7 and Figure 9), suggesting the activation of the JA signaling. In addition, at the last gall information stage, the JA-synthesis-related gene was greatly induced (Figure 7). All of these results suggest that JA signaling might play important role in Chinese chestnuts in response to *D. kuriphilus* infestations.

SA is another plant hormone reported to be involved in host responses to gall formation [18,25]. Consistent with the research on rice gall midge [26], our results also showed that most SA-signaling-related genes were induced, especially during the early gall-formation stage. Plant hormone signal transduction began to show enrichment at stage B (Excel S2), which was consistent with the gall-formation stage. At stage B, the gall tissues continued to grow and expand (Figure 1) and obtain nutrients from the plant host, which induced stress on the chestnut leaves. All of these results strongly suggested that the hormonal signal transduction pathways were utilized to actively combat the infestation of worms.

Apart from hormone signaling, Ca^2+^ signaling was also involved in the plant response to biotic stress [27]. The induction of Ca^2+^ signaling can begin when plants respond to biotic stress [28,29]. Ca^2+^ signaling is involved in the plant defense responses to the bean pyralid (*Lamprosema indicata F.*) in highly resistant soybean [30]. However, Ca^2+^ signaling could play different roles depending on the level of resistance of the plant host to pests. For example, Ca^2+^ signaling can play a negative role in the regulation of defense responses in highly susceptible plant material [30]. Calcium-binding proteins (CMLs) are known for their roles in calcium cell signaling pathways by binding to Ca^2+^ [27]. In addition, *CML* genes in soybeans are modulated in response to bean *L. indicata* larvae [30]. In the present study, ten *CML* genes were downregulated at stage A of the *D. kuriphilus* invasion on the chestnut but upregulated at growth stage B (Figure 7), indicating the transcriptomic response differed at different gall-formation stages. This suggests that Ca^2+^ signaling might be activated in defense against pests when the pest reaches a growth stage. The exact role of Ca^2+^ signaling in the response of chestnuts to *D. kuriphilus* still need further study.

Transcription factors are also involved in plant responses to biotic stress [31]. Agarrwal et al. reported that WRKY transcription factors were induced in rice in response to gall midge attacks [26]. Grunewald et al. reported that the *WRKY23* gene could be induced by *Heterodera schachtii* in *Arabidopsis thaliana* [32]. In this study, we found that 31 *WRKY* genes were significantly differentially expressed after a *D. kuriphilus* infestation (Excel S1). Consistent with previous research [26], most of the *WRKY* transcription factors were upregulated in the chestnuts in response to *D. kuriphilus*. In addition, our qRT-PCR results also confirmed the upregulation of the *WRKY* transcription factors. All of these results suggest that *WRKY* could be induced and be involved in the defense response to gall formation by *D. kuriphilus*.

These signaling or transcription pathways were activated at an early gall-formation stage. They might then regulate downstream defense genes involved in sensing the insect infestation, elevating the basal resistance in chestnuts. This also provides an ideal reference for future research on the defensive mechanism of insects.

### 3.2. Plant Secondary Metabolism Was Altered in Response to Gall Infestation

Pests can manipulate the metabolism of their hosts [17]. Specifically, modulation of plant secondary metabolism has occurred in many insects. Liu et al. reported that Hessian fly *M. destructor* could decrease the concentration of chalcone, isoflanoids, and lignin at the insect feeding site [33]. Similarly, we found that genes involved with secondary metabolism were active during the gall-formation process (Figure 8). Insects can also avoid or suppress the host immune system by regulating plant emissions of volatile compounds that may trigger indirect defenses [13]. Our transcriptomic data showed that *terpene synthases* expression was altered with downregulation either during the whole stage or at the last stage of gall formation (Figure 8). Another gene involved in terpene biosynthesis, *geranylgeranyl diphosphate synthase*, was also downregulated (Figure 8) in chestnuts during gall formation by *D. kuriphilus* (Figure 8). The resistant genotype of *Eucalyptus defensome* had a higher expression of geranylgeranyl phosphate synthase, which suggests the role of this gene in the defense of *E. defensome* to the pest *Leptocybe invasa* [10]. Considering the important role of terpene in plant defenses against insect pests, our results suggest that *D. kuriphilus* might suppress terpene biosynthesis to increase chestnut susceptibility.

Gall-forming insects can alter the defense status in grapes, including increased shikimate and phenylpropanoid biosynthetic pathways, but decreased nonmevalonate and terpenoid biosynthetic pathways [25]. Phenylalanine ammonia lyase (PAL) catalyzes the first step in the phenylpropanoid pathway and is therefore involved in the biosynthesis of polyphenol compounds in plants, such as flavonoids, phenylpropanoids, and lignin [34]. Nabity et al. noted that an increase in lignin-biosynthesis-related genes was beneficial for the development of gall structure formation on plants [25]. Zeng et al. also found that *PAL* genes were up-regulated in soybeans in response to *L. indicata* larvae and suggested that upregulation of the phenylpropanoid biosynthesis pathway might play a role in synthesizing substances to resist insect attacks in soybeans [30]. In this study, the majority of genes associated with the phenylpropanoid biosynthesis pathway, including *PAL*, *CAD*, *CCOAOMT*, *COMT*, and *shikimate O-hydroxycinnamoyltransferase* genes, were upregulated during the gall formation process (Excel S1), which is consistent with previous research [30]. Phenylpropanoids and derivatives might be involved in the chestnut response to insect stress. The exact role of phenylpropanoid metabolism in the interaction of *C. mollissima* BL and *D. kuriphilus* needs further investigation.

Cuticle remodeling and fortification also play crucial roles in plant–pathogen interactions [35]. Previous reports have explored the role of the plant cuticle in changes to sheath epidermal permeability that result from compatible interactions with insect pests [36]. Khajuria et al. found that the expression levels of genes encoding cuticle metabolism-related enzymes were higher, resulting in strengthened cuticles in resistant wheat after Hessian fly infestations [37]. We found that the majority of cutin- and wax-metabolism-related genes were differentially regulated during gall formation of *D. kuriphilus* (Figure 8). In the cuticle biosynthesis process, ECERIFERUM 1 (CER1), which is implicated in alkane formation, was associated with higher resistance of the plant to insect attack [36]. Similarly, higher CER1 might accelerate epicuticular wax synthesis and contribute to the higher resistance of wheat to the Hessian fly [37]. Our results showed that four *CER1* genes were significantly downregulated, especially at early gall stages (Stage A and B). Considering these results, we propose that cuticle metabolism might be affected by *D. kuriphilus* infestations and increase chestnut susceptibility in the initiation and growth stages. Among the genes related to cutin and wax metabolism, we identified cytochrome P450 with more upregulation after *D. kuriphilus* infestation. CYP has an important role in the defense of organisms against pests [38]. Soybean might use the CYP family to mitigate the threat of insect infestation [30]. Hence, we assumed that these differentially expressed genes altogether contributed to the regulation of cuticle metabolism, and then were involved in the response reactions of chestnut to *D. kuriphilus*. KEGG enrichment analysis showed that the secondary metabolic pathway began to show enrichment at stage A (Excel S2). These results suggest that, in the early stages of larval development, regulation of secondary metabolites might be one of the main responses of chestnut. In addition, the significant differences of different gall-formation stages resulted in different transcriptome response of chestnut.

### 3.3. The Stress Response Ability of Chestnut Was Affected by Gall Infestation

Plant defensive responses can be manipulated and reduced by the gall-inducing insects [14]. When attacked by biotrophic pathogens, plants use HR as a strategy to limit the invading pathogen by sacrificing some of their own cells. HR is triggered by an oxidative burst within the cells that releases several reactive oxygen species (ROS) [26]. One example was the presence of a hypersensitive response to *D. kuriphilus* in the hybrid-resistant cultivar ‘Bouche de Bétizac’ (*Castanea sativa* × *Castanea crenata*) [39]. RBOHD-dependent production of ROS was associated with the HR process in plants under biotic and abiotic stresses [40]. In this study, the expression levels of respiratory burst oxidase all increased in response to gall formation by *D. kuriphilus*. Hence, we assumed that HR might also exist in *C. mollissima* BL and that HR might be triggered to increase chestnut resistance against *D. kuriphilus*.

The genes involved in redox, including the major ROS scavenging enzymes, were differentially expressed in rice and allowed effective scavenging of H_2_O_2_ molecules during rice–gall midge interaction [26]. Similarly, we found that genes encoding the CAT enzyme and L-ascorbate peroxidase were upregulated during gall formation in chestnuts (Figure 7). This suggested a decreased accumulation of H_2_O_2_ in chestnut. In contrast, *SOD* genes in this study were almost downregulated. SOD is responsible for catalyzing the dismutation (or partitioning) of the superoxide (O^2−^) radical into either ordinary molecular oxygen (O_2_) or hydrogen peroxide (H_2_O_2_). Therefore, the decrease in *SOD* might also contribute to the decreased content of H_2_O_2_ in chestnuts during gall formation. Laloi et al. reported that the overexpression of ascorbate peroxidase (H_2_O_2_ scavenger) enhanced the intensity of the stress response that was mediated by a singlet oxygen in *Arabidopsis* [41]. Thus, we believe that HR is not triggered by H_2_O_2_ but by other ROS, and this is consistent with the rice–gall midge interaction [26]. An increase in catalase activity can be associated with the defensive response of the host-plant against insect activity [7]. In addition to *SOD* and *CAT*, *peroxidase* has also been reported to be associated with plant responses to insects [42,43]. Our results showed that the majority of peroxidases in chestnuts were induced by *D. kuriphilus* (Figure 7).

Plants can produce a variety of defense proteins, including PR, proteinase inhibitors, chitinases, and lectins, to resist insect pests [30]. Pathogenesis-related proteins have established roles in the defense mechanism of plants [44]. After a *D. kuriphilus* infestation, three genes encoding pathogenesis-related proteins were significantly induced during the entire gall formation process, and two were upregulated at stage D (Excel S1). PRs were also induced by *L. indicata* larvae in soybeans and might be involved in the defense responses of soybeans to *L. indicata* [30]. All of the chitinase genes identified in this study were also upregulated after a *D. kuriphilus* infestation, especially at the late stage of gall formation (Excel S1). Zeng et al. demonstrated that the chitinase gene could also be induced by *L. indicata* [30]. Based on these results, we believe that the genes encoding PR and chitinase might be involved in the defense responses of chestnuts to *D. kuriphilus*.

In summary, our data demonstrated that a variety of response reactions were influenced in Chinese chestnuts after *D. kuriphilus* infestation.

## 4. Materials and Methods

### 4.1. Plant Material and Gall Collection

One-year-old Chinese chestnut (*Castanea mollssina* Blume) plants ‘Hongli’ were planted in Chinese chestnut germplasm resources, Nanjing City, Jiangsu Province, China (32°06′ N, 118°84′ E, altitude: 240 m), in April 2017. Leaf samples together with gall were collected at four gall stages (Initiation (A) at April 7, Early growth (B) at April 10, Late growth (C) at April 15, and Maturation (D) at April 26) in 2017. A healthy leaf was used as a control. Each sample contained three biological replicates. The fresh leaves were harvested, frozen in liquid nitrogen, and stored at −80 °C until use.

### 4.2. RNA Extraction, Library Construction, and Sequencing

Total RNA was isolated using the Column Plant RNA Out kit (Fuji, China). The quantity and quality of the RNA samples were verified using a NanoDrop2000 Spectrophotometer (Thermo Fisher Scientific, Waltham, MA, USA), agarose gel electrophoresis (1.0%), and an Agilent 2100 Bioanalyzer (Agilent Technologies, Inc., Santa Clara, CA, USA). Satisfactory RNA with an RNA Integrity Number (RIN) greater than 7.0 was used for library construction and sequencing. The detailed information for total RNA quality and concentration was shown in Excel S3. A total of 12 libraries were constructed and sequenced with SE 50 using the Illumina Hiseq2500 platform according to standard procedures. Each sample contained three biological replicates. All of the sequencing data were submitted to the NCBI Sequence Read Archive under BioProject ID PRJNA512447.

### 4.3. De Novo Assembly and Annotation of the Castanea mollissima BL unigenes

Clean data were obtained from raw data by removing adapter sequences and trimming reads with poly-N and low quality reads (the percentage of low quality bases is over 50% in a read). Clean reads from all samples were mapped to a *C. mollissima* Bl. reference genome (https://www.hardwoodgenomics.org/Genome-assembly/1962958) by using HISAT2 software [45] for further analysis. The number of fragments per kb per million reads (FPKM) method was applied to calculate the gene expression level.

### 4.4. Differential Expressed Genes (DEGs) Analysis

The NOISeq method was used to obtain the “base mean” value for identifying DEGs [46]. Foldchange ≥2 and divergence probability ≥0.8 were set as the thresholds for the significance of the gene expression difference between the two samples.

All of the mapped genes were compared with public databases, including the NR database (http://www.ncbi.nlm.nih.gov/), Swiss-Prot database (http://www.expasy.ch/sprot), the COG (http://www.ncbi.nlm.nih.gov/COG/), Pfam (http://pfam.xfam.org/) databases, by using BLASTX (http://blast.ncbi.nlm.nih.gov/Blast.cgi) with an E-value of 1e-5, and searched against the Pfam database (http://pfam.xfam.org/) by hmmscan (v3.0) software [47]. Then, WEGO software was used to obtain the Gene Ontology (GO) functional classifications for all of the annotated genes [48]. A Python script was used to retrieve Kyoto Encyclopedia of Genes and Genomes (KEGG) information from the blast result, establish pathway associations between annotated genes and the database, and draw the KEGG classification map. GO enrichment analyses of DEGs were performed using the singular enrichment analysis (SEA) method with *p* < 0.01 and FDR < 0.05 by agriGO [49]. The hypergeometric Fisher exact test (*p* < 0.05) and Benjamini (FDR < 0.05) were performed to detect statistically significant enrichment of KEGG pathways. GO and KEGG enrichment analyses were performed using the whole eggplant transcriptome setting as the background. Transcription factors were identified using PlantTFDB 4.0 with default parameters [50]. Heatmaps of the gene sets were analyzed using R package ‘pheatmap’ (https://github.com/raivokolde/pheatmap). The gene expression levels were transformed by log2 (mean of FPKM+1) using three biological replications.

### 4.5. Validation of DEGs by Quantitative Real-Time PCR (qRT-PCR)

Total RNA was isolated as described above. The first-strand cDNA was synthesized from 2 μg of total RNA using a Prime Script RT Reagent Kit (Takara, Kusatsu, Japan). The qRT-PCR reactions were performed in 96-well plates using the ABI7500 fast Real-Time PCR system (Applied Biosystems, Foster City, CA, USA) with the SYBR Premix ExTaq™ Kit (Takara, Dalian, China). The *Actin* gene was used as an internal control due to its stable expression during gall-formation stages (Table S2). The relative expression levels were calculated using the 2^−ΔΔ*C*t^ method [51]. Three biological replicates were performed. The primers were designed using NCBI primer-BLAST (https://www.ncbi.nlm.nih.gov/tools/primer-blast/). Detailed information on the genes and primer sequences are listed in Table S3.

### 4.6. Statistical Analyses

All experiments were conducted with three biological replicates. Data analyses were conducted using SPSS version 17.0 statistical software.

## 5. Conclusions

We investigated the molecular basis of Chinese chestnut responses to *D. kuriphilus* infestations via transcriptomic analysis of DEGs during different gall-formation stages. In response to insect attack, plant hormone signaling, transcription, and Ca^2+^-mediated signal transduction pathways, secondary metabolism and stress responses occurred in the Chinese chestnut. DEGs involved in these biological processes might represent Chinese chestnut defense genes. The study data provides new information on important molecular level pathways used by *C. mollissima* during *D. kuriphilus* gall formation.

## Supplementary Materials

Supplementary materials can be found at http://www.mdpi.com/1422-0067/20/4/855/s1.

## Abbreviations

DEGs	Differentially expressed genes
ROS	Reactive oxygen species
KEGG	Kyoto Encyclopedia of Genes and Genomes
GO	Gene ontology
SOD	Superoxide dismutase
CAT	Catalase
BH	Benjamini and Hochberg
FDR	False discovery rate
