# Supplementary material for: Transcriptome Analysis of Chinese Chestnut (Castanea mollissima Blume) in Response to Dryocosmus kuriphilus Yasumatsu Infestation
Source: Int J Mol Sci. 2019 Feb 15;20(4):855. doi: 10.3390/ijms20040855 (PMC6412832; doi:10.3390/ijms20040855)

Supplementary Material Figure S2. Spearman correlation analysis of the three replicates in each group. The vertical and horizontal axes represent the expression levels of all unigenes (log10 (FPKM)).


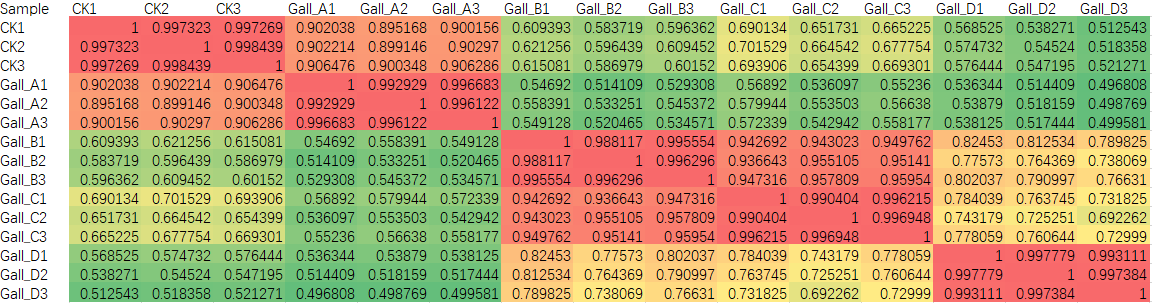

Supplement: Supplementary file 1 [file ijms-20-00855-s001.zip › Supplementary Figure S2.docx]
