# Supplementary material for: Transcriptome Analysis of Chinese Chestnut (Castanea mollissima Blume) in Response to Dryocosmus kuriphilus Yasumatsu Infestation
Source: Int J Mol Sci. 2019 Feb 15;20(4):855. doi: 10.3390/ijms20040855 (PMC6412832; doi:10.3390/ijms20040855)
Supplement: Supplementary file 1 [file ijms-20-00855-s001.zip › Supplementary Table S1.docx]

Supplementary Material Table S1. Summary of RNA-Seq data.

| **Sample** | **Raw Data Size (bp)** | **Raw Reads Number** | **Clean Reads Number** | **Clean Data Rate (%)** | **Clean Read Q20(%) >= 90** | **Unique Match(%)** |
| --- | --- | --- | --- | --- | --- | --- |
| CK1 | 1,206,820,450 | 24,136,409 | 24,053,741 | 99.65 | 98.3 | 65.52 |
| CK2 | 1,203,789,000 | 24,075,780 | 23,895,516 | 99.25 | 98.4 | 64.85 |
| CK3 | 1,204,162,900 | 24,083,258 | 23,925,579 | 99.34 | 98.2 | 65.42 |
| Gall_A1 | 1,206,828,050 | 24,136,561 | 23,961,495 | 99.27 | 98.2 | 65.78 |
| Gall_A2 | 1,203,464,200 | 24,069,284 | 23,906,005 | 99.32 | 98.2 | 64.43 |
| Gall_A3 | 1,203,966,550 | 24,079,331 | 23,866,800 | 99.11 | 98.3 | 65.34 |
| Gall_B1 | 1,206,812,950 | 24,136,259 | 24,065,260 | 99.7 | 98.4 | 65.46 |
| Gall_B2 | 1,203,728,700 | 24,074,574 | 23,904,252 | 99.29 | 98.2 | 64.13 |
| Gall_B3 | 1,204,500,900 | 24,090,018 | 23,897,240 | 99.19 | 98.3 | 64.39 |
| Gall_C1 | 1,206,851,050 | 24,137,021 | 24,048,142 | 99.63 | 98.2 | 64.65 |
| Gall_C2 | 1,204,122,000 | 24,082,440 | 23,921,578 | 99.33 | 98.4 | 63.64 |
| Gall_C3 | 1,204,337,400 | 24,086,748 | 23,876,167 | 99.12 | 98.3 | 64.09 |
| Gall_D1 | 1,206,828,150 | 24,136,563 | 24,006,261 | 99.46 | 98.3 | 59.90 |
| Gall_D2 | 1,204,203,050 | 24,084,061 | 23,883,321 | 99.16 | 98.3 | 59.63 |
| Gall_D3 | 1,204,603,900 | 24,092,078 | 23,926,580 | 99.31 | 98.4 | 60.57 |
| Average | 1,205,001,283 | 24,100,026 | 23,942,529 | 99.34 | 98.29 | 63.85 |
| Total | 18,075,019,250 | 361,500,385 | 359,137,937 |  |  |  |
