# Supplementary material for: Transcriptome Analysis of Chinese Chestnut (Castanea mollissima Blume) in Response to Dryocosmus kuriphilus Yasumatsu Infestation
Source: Int J Mol Sci. 2019 Feb 15;20(4):855. doi: 10.3390/ijms20040855 (PMC6412832; doi:10.3390/ijms20040855)
Supplement: Supplementary file 1 [file ijms-20-00855-s001.zip › Supplementary Table S2.docx]

Supplemental Table S2. The expression of CmACT1 during gall formation stages.

| gene | HL_CK1_FPKM | HL_CK2_FPKM | HL_CK3_FPKM |
| --- | --- | --- | --- |
| CmACT1 | 766.69 | 787.37 | 764.3 |
| gene | HL_Gall_A1_FPKM | HL_Gall_A2_FPKM | HL_Gall_A3_FPKM |
| CmACT1 | 775.72 | 725.58 | 764.8 |
| gene | HL_Gall_B1_FPKM | HL_Gall_B2_FPKM | HL_Gall_B3_FPKM |
| CmACT1 | 719.63 | 725.71 | 717.47 |
| gene | HL_Gall_C1_FPKM | HL_Gall_C2_FPKM | HL_Gall_C3_FPKM |
| CmACT1 | 741.59 | 753.47 | 771.48 |
| gene | HL_Gall_D1_FPKM | HL_Gall_D2_FPKM | HL_Gall_D3_FPKM |
| CmACT1 | 623.76 | 626.57 | 679.94 |
