# Supplementary material for: Transcriptome Analysis of Chinese Chestnut (Castanea mollissima Blume) in Response to Dryocosmus kuriphilus Yasumatsu Infestation
Source: Int J Mol Sci. 2019 Feb 15;20(4):855. doi: 10.3390/ijms20040855 (PMC6412832; doi:10.3390/ijms20040855)
Supplement: Supplementary file 1 [file ijms-20-00855-s001.zip › Supplementary Table S3.docx]

Supplementary Material Table S2. The primer sequences of 19 unigenes for qRT-PCR in this study.

| gene | Unigene ID | Forward primer (5'→3') | Reverse primer (5'→3') |
| --- | --- | --- | --- |
| *WRKY27* | maker-scaffold01178-augustus-gene-0.35 | CAGCCAACCACAACATCAAC | TCATCCTCATCATCATCAACCATT |
| *WRKY75* | maker-scaffold04189-snap-gene-0.19 | TTCATCTTCAACACCACCAACTT | GCTTCTGCTCTGAGGAACATC |
| *WRKY1* | maker-scaffold00281-snap-gene-0.36 | GTTCATCAGCAGTTTCTACTTCCT | GTCCTTCTTGTTGGCACCAT |
| *WRKY65* | maker-scaffold12041-augustus-gene-0.11 | TGCGTCAATGCTCATCATCAC | TGCTAGTTGGATCAGGTTGGT |
| *WRKY31* | maker-scaffold05412-augustus-gene-0.21 | ATTATTGGTGGTGGTGCTCATC | CATTGCTGTTGGAGGTGGTAG |
| *bHLH93* | maker-scaffold11285-snap-gene-0.6 | ACCACACTTGATACACCACCTT | TGGAGACTGAATTGGCTCTACTT |
| *Respiratory burst oxidase* | augustus_masked-scaffold00111-abinit-gene-1.1 | ACAATCCACTCCTCCAACTCA | TCAACGGCAGACCATCCA |
| *NPR3-like* | maker-scaffold01318-augustus-gene-0.34 | TAAGCCATCGCCACCAGAA | AGCATACATCAATTCCACAGCATA |
| *NPR5* | maker-scaffold02523-snap-gene-0.12 | CTCATCACCACCATCACCAT | TTCACAACTTCTCGGCTACAAT |
| *NPR3* | maker-scaffold08413-augustus-gene-0.8 | GTCCTCCGCCTCCTTCTG | ACAACCGCATCGCTATAATCAC |
| *NPR1* | maker-scaffold03463-snap-gene-0.18 | AACGCCTTCTCCAATTCCAA | TCTGACCTTGCCGCTATACA |
| *bHLH93* | maker-scaffold04445-augustus-gene-0.11 | GCACACCATTGAAGCACTAGG | TCTTCTACAGACATCTTCCACCAT |
| *bHLH* | maker-scaffold00859-augustus-gene-0.50 | TGATAACAACAGCAAGGACAATG | ACGCCCACTTCAGTTTCTTT |
| *bHLH61* | maker-scaffold01868-augustus-gene-0.12 | CACTCTTTGCTTCTTCCTCTTCT | TGGCTAATTGGCTTACTATGTTCT |
| *Pathogenesis-related protein 1* | maker-scaffold09767-snap-gene-0.8 | GCATTCAGGAGGACCTTACG | GCACTCACCACCAACACAT |
| *GDSL esterase/lipase* | maker-scaffold03374-snap-gene-0.22 | ATAAGTCGCATACCGTTCAATCAA | CCGAGCCACATCATCAGC |
| *Calcium-binding protein CML* | maker-scaffold00418-augustus-gene-0.47 | TAAGCGAAGTTGATGTTGATGGAA | CATTACATGCCTCAGCTCAGTAG |
| *Catalase1* | snap_masked-scaffold00568-abinit-gene-0.13 | GCACCAGACAGGCAAGAG | TGAGACCAGTATGAGATCCAGATG |
| *Catalase* | maker-scaffold00568-snap-gene-0.19 | TCCACAAGACTACAGGCACAT | ATTCGTTCCTCCAATCCTAATAGC |
| *CmACT1* |  | TTGACTATGAGCAGGAACTT | TTGTAGGTGGTCTCGTGAAT |
