# Supplementary figures and images for: Transcriptome Analysis of Chinese Chestnut (Castanea mollissima Blume) in Response to Dryocosmus kuriphilus Yasumatsu Infestation
Source: Int J Mol Sci. 2019 Feb 15;20(4):855. doi: 10.3390/ijms20040855 (PMC6412832; doi:10.3390/ijms20040855)

Supplementary Material Figure S1. The saturation curves of 15 RNA-seq samples.


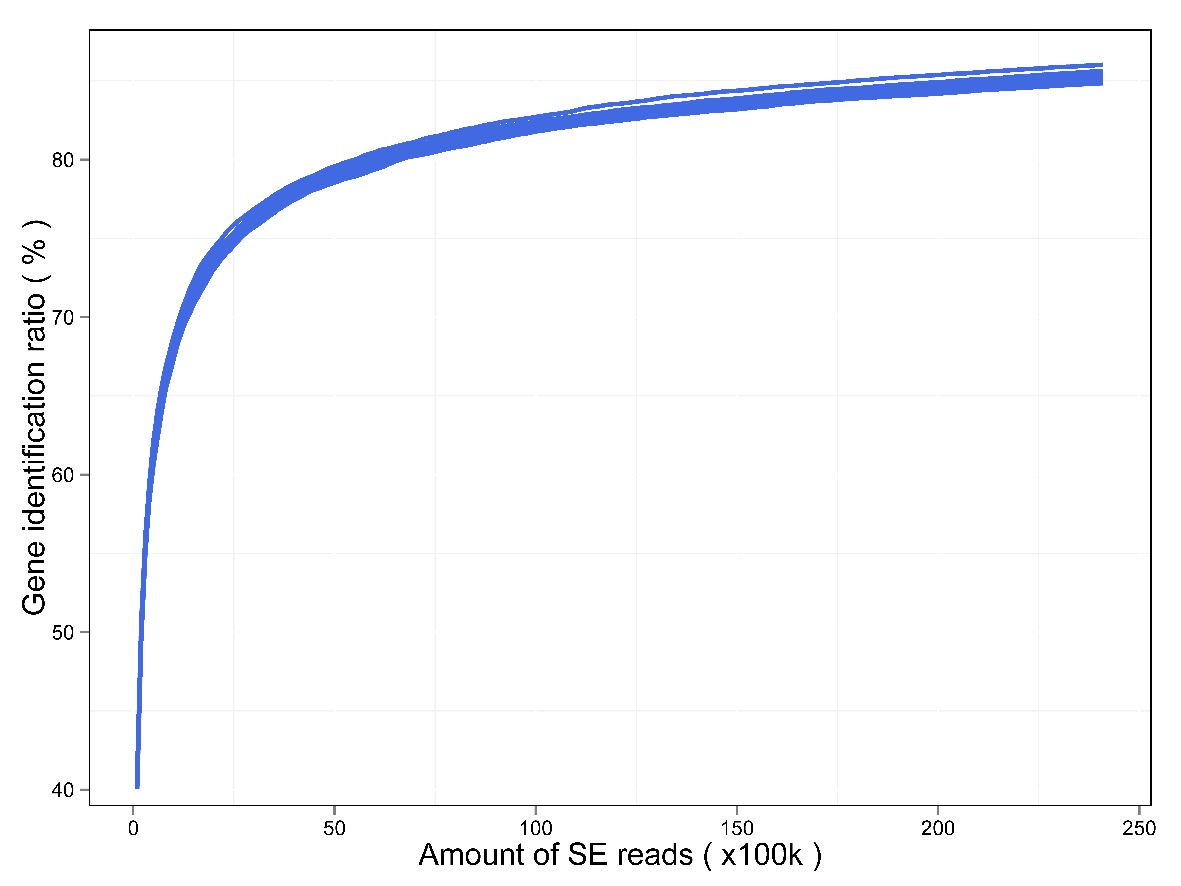

Supplement: Supplementary file 1 [file ijms-20-00855-s001.zip › Supplementary Figure S1.docx]
